# Adipokines and Non-Alcoholic Fatty Liver Disease: Multiple Interactions

**DOI:** 10.3390/ijms18081649

**Published:** 2017-07-29

**Authors:** Timon E. Adolph, Christoph Grander, Felix Grabherr, Herbert Tilg

**Affiliations:** Department of Internal Medicine I, Gastroenterology, Hepatology & Endocrinology, Medical University Innsbruck, A-6020 Innsbruck, Austria; timon-erik.adolph@i-med.ac.at (T.E.A.); christoph.grander@i-med.ac.at (C.G.); felix.grabherr@i-med.ac.at (F.G.)

**Keywords:** Adipokines, hepatocellular cancer, metabolism, metabolic inflammation, non-alcoholic fatty liver disease (NAFLD)

## Abstract

Accumulating evidence links obesity with low-grade inflammation which may originate from adipose tissue that secretes a plethora of pro- and anti-inflammatory cytokines termed adipokines. Adiponectin and leptin have evolved as crucial signals in many obesity-related pathologies including non-alcoholic fatty liver disease (NAFLD). Whereas adiponectin deficiency might be critically involved in the pro-inflammatory state associated with obesity and related disorders, overproduction of leptin, a rather pro-inflammatory mediator, is considered of equal relevance. An imbalanced adipokine profile in obesity consecutively contributes to metabolic inflammation in NAFLD, which is associated with a substantial risk for developing hepatocellular carcinoma (HCC) also in the non-cirrhotic stage of disease. Both adiponectin and leptin have been related to liver tumorigenesis especially in preclinical models. This review covers recent advances in our understanding of some adipokines in NAFLD and associated HCC.

## 1. Introduction

Obesity and related disorders are critically emerging worldwide, such that an epidemic has been proclaimed [1,2]. It is conceived that metabolic dysfunction in adipose tissue promotes immune dysregulation which governs metabolic diseases [3]. In this context, adipokines secreted by adipose tissue (i.e., adipocytes, stromal and/or immune cells) have gained attention over the last decade as they fuel metabolic dysfunction [4,5,6,7,8,9]. Visceral and subcutaneous adipose tissues are the most abundant fat depots with distinct adipokine profiles [10]. Adipokines comprise a heterogeneous family of cytokines as recently reviewed [9]. The first adipokine identified was Adipsin in 1987 followed by TNFα and Leptin; since then, a plethora of adipose tissue mediators has been described [9,11]. While most adipokines are upregulated in obesity and promote inflammatory responses (e.g., Leptin, TNFα, IL-6, and IL-18), others may act as anti-inflammatory modulators (e.g., adiponectin, and secreted frizzled related protein 5). These observations formed the basis for a model in which adipokine imbalance promotes susceptibility to metabolic and vascular diseases in obesity [3,9,12].

The liver closely communicates with adipose tissue [13]. NAFLD, the hepatic manifestation of the metabolic syndrome, comprises a spectrum of liver diseases including benign steatosis, steatohepatitis, cirrhosis and hepatocellular carcinoma. The pathophysiology of NAFLD involves gut-derived microbial components, lipotoxicity and inflammation which may occur in the liver, but may also originate from other tissues such as adipose tissue or the gastrointestinal tract [5]. Adipose tissue inflammation is affected by adipokines which have a demonstrable impact on NAFLD by regulation of hepatic fat accumulation, insulin resistance and fibrosis. As such, the combination of clinical data and basic research may elegantly explain how adipokines in obesity promote the evolution of NAFLD.

## 2. Adiponectin and Leptin: The Two Major Players

### 2.1. Adiponectin

Adiponectin is an adipocyte-derived anti-inflammatory mediator that acts via two receptors (ADIPOR1 and ADIPOR2) that elicit AMP kinase signaling [14], and might be modulated by T-cadherin [15,16]. Adiponectin is detectable in the circulation in various isoforms either as a full-length (low molecular weight, medium and high molecular weight isoform) or a smaller, globular fragment. Adiponectin suppresses adipose TNFα expression and induces anti-inflammatory gene expression in human leukocytes including IL-10 and IL-1 receptor antagonist [17,18]. The anti-inflammatory activities of adiponectin also involve other pathways such as induction of heme-oxygenase-1 (HO-1). Blockade of HO-1 activity prevents the inhibitory effect of adiponectin on LPS-stimulated TNFα expression [19]. When mice were fed cobalt protoporphyrin to induce HO-1 expression and thereby activate the IL-10/STAT3/HO-1 pathway, ethanol-induced sensitivity to LPS was improved. Adiponectin also protects against iron-induced liver injury by up-regulation of HO-1 [20].

Adiponectin exerts many of its functions via two distinct receptors (ADIPOR1 and ADIPOR2), both seven-transmembrane domain proteins with an internal *N*-terminus part and an extracellular *C*-terminus part [21,22]. Genetic deletion of ADIPOR1 and ADIPOR2 in a mouse model resulted in metabolic dysfunction [23]. Convincing evidence for an anti-inflammatory activity of adiponectin came from studies in adiponectin-deficient mice [17]. In these studies, adiponectin knockout (KO) mice showed high levels of TNFα mRNA expression in adipose tissue and high TNFα protein concentrations in the circulation. Weight loss itself is also a potent inducer of adiponectin synthesis [24]. Serum levels of adiponectin are extremely well studied and it has been very convincingly demonstrated that in obesity and its related disorders adiponectin serum levels are reduced [4,25], while certain drugs such as thiazolidinediones/PPARγ activation induce adiponectin [26]. Probably the most compelling evidence for a predominant role of this mediator in obesity and related disorders has been derived from studies generating adipose tissue-specific adiponectin transgenic animals [27]. These mice, despite becoming extremely obese, were metabolically healthy.

Adipose tissue is the major site of endogenous adiponectin production, even though other potential sources such as muscle cells, cardiac myocytes or endothelial cells have also been identified [28]. In addition, with respect to biological function, adipose tissue-derived adiponectin might play an overwhelming role. This finding is substantially supported by studies generating adipose-tissue specific KO mice [29]. In this study, selective deletion of conventional kinesin heavy chain (Kif5b) in adipose tissue, which mediates adiponectin secretion, exacerbates high-fat diet (HFD)-induced obesity and its associated metabolic disorders. This phenotype was characterized by a decrease in energy expenditure, suppression of adiponectin secretion, an increase in circulating leptin levels, and impaired insulin signaling. Inflammation in general also negatively affects adiponectin synthesis as TNFα suppresses the transcription of adiponectin in 3T3-L1 adipocytes, which might explain the lower adiponectin mRNA levels in obesity.

#### Adiponectin and Non-Alcoholic Fatty Liver Disease

Adiponectin reflects the best studied adipokine in NAFLD and findings are rather consistent compared to other adipokines as discussed later. Plasma levels of adiponectin are markedly diminished in visceral obesity and states of insulin resistance such as non-alcoholic steatohepatitis (NASH), atherosclerosis and type 2 diabetes mellitus [30,31]. We previously showed decreased hepatic adiponectin expression in patients with NASH and enhanced liver expression of adiponectin and its specific receptors after successful weight loss [32]. The association between NAFLD and serum levels of adiponectin has been extensively studied in the last years and recently analyzed in detail by Polyzos and colleagues [33]. This meta-analysis included 28 studies on 2243 subjects (698 controls, 1545 patients with NAFLD) and a substantial group had also undergone liver biopsy. Hypoadiponectinemia was especially a feature of patients with NASH. It is still unclear why transition from simple fatty liver to NASH results in a further decrease in systemic adiponectin levels. We have shown that high molecular weight (HMW) rather than total adiponectin was correlated with extent of liver steatosis assessed by sonography before and six months after bariatric surgery [34]. Gender-specific issues are also of interest as shown from the RAINE Study from Western Australia where males showed reduced serum adiponectin concentrations compared to females [35]. A major role for the relevance of serum adiponectin assessment as prognostic factor in NAFLD has, however, been questioned by several large prospective studies [36,37]. Despite the large number of studies available with a focus on serum adiponectin levels and NAFLD, specific issues such as the role of various isoforms need to be addressed in more detail in the future. Various potential NASH therapies such as PPARγ agonists or vitamin E are able to-up-regulate adiponectin level besides many other effects [38,39].

Importantly, and thereby highlighting a key role for adiponectin in NAFLD, it could be recently demonstrated that lean NAFLD patients show reduced circulating adiponectin concentrations [40]. An important role for adiponectin in NASH has also recently been derived from an animal model: Deletion of *C*-terminus Hsc70-Interacting Protein (CHIP) caused oxidative stress insulin resistance and hepatic inflammation in mice, however the authors noted little evidence for NAFLD after eight months. One explanation for the absence of a NAFLD phenotype may be a compensatory upregulation of adiponectin which activates the AMPK–FOXO-signaling axis and may override detrimental oxidative stress and JNK signaling [41]. Overexpression of adiponectin was responsible in counteracting progression of hepatic microvesicular steatosis. As such, this study and others highlight a key role for adiponectin as protective metabolic player in obesity and related disorders.

### 2.2. Leptin

The role of leptin in modulating immunity and inflammation has become increasingly evident in the last years [42]. Besides regulating neuroendocrine function, energy homeostasis, hematopoiesis and angiogenesis, leptin is an important mediator of immune-mediated diseases and inflammatory processes [43]. In addition, leptin has rather pro-inflammatory functions in various models of auto-inflammatory or immune-mediated inflammatory disorders [44]. Leptin expression is regulated by insulin and glucocorticoids, is expressed predominantly in adipose tissue and secreted into the circulation [45]. Leptin mediates adipose-brain communication and regulates appetite by targeting leptin receptor type B (LEPRb)-expressing neurons in the hypothalamus. LEPRb is an IL-6 like receptor that signals via JAK2 thereby activating STAT transcription factors [45]. By these means, leptin induces the expression of inflammatory cytokines which may in turn release leptin from adipocytes [46]. Importantly, in the steady state, leptin appears to improve metabolic dysregulation. In obesity, however, leptin fails to correct hyperglycemia, such that it is conceived that metabolic dysregulation and obesity may cause “leptin resistance” [47]. Leptin resistance or deficiency might be overcome by certain adipose tissue-derived factors such as fibroblast growth factor 1 (FGF1). Administration of FGF1 in models of NAFLD, namely in *ob*/*ob* mice and in choline-deficiency, ameliorates hepatic steatosis and therefore this factor could not only act as potent glucose-lowering and insulin-sensitizing agent but also beneficially regulate hepatic lipid metabolism [48].

#### Leptin and NAFLD

Results obtained from leptin NAFLD studies are more controversial and heterogeneous compared to those with adiponectin. We have provided data that leptin mRNA expression and immunostaining in the liver remained stable after six months of massive weight loss [32]. This, however, might not rule out that sources other than the liver are responsible for the sometimes observed decrease in serum leptin levels after bariatric surgery [49]. Increased serum leptin levels have also been correlated with severity of liver disease i.e., the amount of inflammation and fibrosis [50]. Increased serum leptin levels were also observed in other large prospective NAFLD studies [36,37]. Serum leptin concentrations demonstrated an association with NAFLD both in male and female pre-diabetic subjects and this association was mediated by insulin secretory dysfunction and insulin resistance [51]. Certain polymorphisms might also be associated with metabolic liver disease, as demonstrated by a recent study from China. Here, LEPR Q223R polymorphisms may confer a significant risk of NAFLD and coronary atherosclerosis [52]. Metformin, although not proven as an effective therapy in human NASH, is able to upregulate leptin receptor expression in mice paralleled by decreased hepatic triglyceride levels [53]. An increase of soluble leptin receptors was also observed in type 2 diabetes patients after metformin treatment. A recent meta-analysis by Polyzos and colleagues has nicely summarized the current status of leptin in NAFLD [54]. In summary, 33 studies with 2612 individuals were analyzed. Patients with simple steatosis and NASH exhibited higher serum levels of leptin compared to controls and high leptin concentrations were associated with increased severity of NAFLD. To conclude, an enormous number of clinical studies have well established serum profiles of adiponectin and leptin in human NAFLD. These studies demonstrated that adiponectin concentrations are decreased while leptin levels increased in NAFLD suggesting that a dysbalance of adipokines might promote evolution of this systemic disease.

## 3. Adiponectin and Leptin: Potential Relevance in Hepatocellular Carcinoma (HCC) Associated with NAFLD

Because of the strong association of HCC with obesity it appears plausible that adipokines might play a role in NAFLD-associated HCC. It has been recognized in the past years that NAFLD exerts a substantial risk for the development of hepatocellular carcinoma [55,56] which turned out to be of great clinical relevance as this association has also been observed in the non-cirrhotic stage. This raises the possibility that a fatty liver *per se* enriched with various inflammatory mediators such as adipokines might reflect a driving force in this entity. However, it is noteworthy that several other classical pro-inflammatory cytokines expressed either in adipose tissue or the liver (e.g., TNFα or IL-6) are likely candidates to play a role in the chronic inflammatory state which promotes tumor evolution [57,58]. This is of clinical relevance in severe obesity as IL-6 is highly expressed both, in liver and adipose tissue, and successful weight loss as achieved by bariatric surgery almost eliminates this overproduction [24].

### 3.1. Adiponectin and HCC

Advanced liver diseases are associated with increased serum adiponectin levels [59]. Cirrhotic and non-cirrhotic HCC patients demonstrated increased serum levels of both adiponectin and leptin [60]. In chronic hepatitis B, patients with cirrhosis and HCC also demonstrate markedly increased adiponectin levels [61] including increased expression in HCC tissue samples [62]. High adiponectin serum levels might also predict the consecutive development of HCC [63]. Patients with increased serum levels of adiponectin had an increased risk for HCC development in subsequent disease course [64]. Higher plasma levels of adiponectin could predict poor HCC survival among patients without liver transplantation [65,66]. Higher levels of non-HMW adiponectin also conferred an increased risk for later development of HCC in a large prospective study [67].

A role for adiponectin in liver tumor formation is also supported by preclinical studies. Hypoadiponectinemia promotes liver tumor formation in a choline-deficient mouse NASH model [68]. After 24 weeks, knockout mice developed liver cirrhosis and hepatic tumors, whereas wild-type mice exhibited simple steatosis. Mechanistically, adiponectin has been shown to increase apoptosis of HCC cells through activation of caspase-3, and increased phosphorylation of c-Jun *N*-terminal kinase (JNK). Inhibition of JNK-phosphorylation prevented this apoptotic effect of adiponectin [69]. Adiponectin expression in a tissue microarray of human HCC inversely correlated with tumor size. It may be speculated that adiponectin and its interaction with receptors might exert anti-tumor activities. Adiponectin exhibited chemoprotective and hepatoprotective functions by blocking sulfatase 2 [70]. However, the role of adiponectin in HCC is incompletely understood.

### 3.2. Leptin and HCC

Whereas levels of non-HMW adiponectin evolved as an attractive biomarker in predicting later development of HCC in a large prospective study, results for serum leptin were negative [67]. In addition, in the other aforementioned study in which adiponectin strongly correlated with stage of liver disease, presence of metastasis, α-fetoprotein (AFP) and Barcelona clinic liver cancer (BCLC) stage B/C and survival, no significant impact was observed for leptin on HCC survival [65]. Adiponectin treatment suppresses leptin-induced cell proliferation of HCC cells and adiponectin treatment impairs leptin-induced invasion of HCC cells [71]. In human HCC samples, leptin expression was associated with HCC proliferation as evaluated by Ki-67, whereas adiponectin expression correlated significantly with increased disease-free survival and inversely with tumor size and local recurrence [71]. Increased leptin expression in HCC tissue has also been observed by other investigators and leptin expression was related to the expression of human telomerase reverse transcriptase [72]. In tissue samples derived from human HCC and in hepatoma cell lines, a substantially higher production of leptin has been observed [73]. Interestingly, in this study hepatoma cells enhanced anti-HCC immunity through secretion of leptin resulting in down-regulation of Treg activity and subsequent promotion of CD8+ T-cell responses. Analysis of liver tissue samples from HCC patients exhibit somatic mutations in the leptin receptor (LEPR) in the stage of cirrhosis during chronic HCV infection [74]. These mutations were able to disrupt LEPR signaling and increase susceptibility to hepatocarcinogenesis. Moreover, 40% of LEPB-deficient (C57BL/KsJ-db/db) mice exhibited liver tumors induced by thioacetamide suggesting a role for the leptin pathway in hepatic tumorigenesis. Major alterations in cytokine profiles in the plasma and liver tissue lysates from normal and steatotic mice have been identified including leptin, CXCL1, CXCL2, and CXCL16 which exhibit proliferative functions in vitro [75]. In conclusion, only few studies have assessed serum levels of leptin in HCC, and these have revealed rather negative results. Some studies assessing HCC in human tissue have provided evidence that leptin could play a role in obesity-related tumorigenesis. As such, adipokines including adiponectin and leptin represent key players in obesity-related disorders and might be involved in the pathogenesis of NAFLD and HCC. Further studies are needed to understand their role in malignant disease processes.

## 4. Other Adipokines

In obesity, adipose tissue represents a rich source for mediators, which supposedly act systemically and influence pathways throughout the organism mainly in a detrimental manner. In the past years many such mediators have been identified and for most of them clinical relevance until today remains unclear. As this article has a focus on adiponectin and leptin, probably the most relevant adipose tissue-derived mediators currently known, we only briefly discuss a few other potential players and their role in NAFLD as this topic has been excellently reviewed recently [7].

### 4.1. NAMPT/Visfatin

Pre-B cell colony enhancing factor (PBEF) (also called nicotinamide phosphoribosyl transferase, NAMPT or visfatin) is produced by many cell types throughout the body. Although initially described as a cytokine, its rediscovery as the key enzyme in nicotinamide adenine dinucleotide (NAD) generation has considerably changed its biological perspective. Both extracellular (cytokine-like) and intracellular (enzymatic) functions are responsible for its relevance in immune, metabolic and stress responses. As a signaling molecule, NAMPT mainly acts pro-inflammatory by inducing cytokines such as tumor necrosis factor α (TNFα) and interleukin-6 (IL-6) [76]. Patients with NAFLD exhibit increased serum concentrations of NAMPT and weight loss is associated both with a decrease in serum levels and reduced liver expression. Liver immunohistochemistry performed on baseline and follow-up liver biopsies demonstrated a decrease in NAMPT immunoreactivity [32]. In a case-controlled study of 70 human NAFLD subjects, serum levels of NAMPT, IL-8 and TNFα were positively correlated with the presence of NASH [77]. NAMPT modulates various pathways in obesity and related disorders such as NAFLD affecting oxidative stress response, apoptosis, lipid and glucose metabolism, inflammation and insulin resistance [78]. Although NAMPT also has a crucial role in cancer cell metabolism, a specific role in HCC associated with NAFLD has never been demonstrated. Despite several studies the role of NAMPT in human NAFLD remains inconclusive and recently it has been demonstrated that inhibition of NAMPT aggravated the HFD- or oleic acid-induced hepatic steatosis through suppression of Sirt1-mediated signaling pathways [79]. The interaction with Sirt1 might be relevant, as we have shown earlier that weight loss after bariatric surgery is able to induce expression of Sirt1 and -3, in both adipose and liver tissue [80].

### 4.2. Resistin

The initial discovery of resistin suggested a major role for this mediator in diabetes and insulin resistance [81]. This study in rodents showed that resistin is primarily produced in adipose tissue and/or by adipocytes and is involved in insulin resistance. Targeting resistin by specific antibodies improved metabolic dysregulation. Its role in human diseases associated with obesity such as NAFLD remains, however, unclear. Studies using human peripheral blood mononuclear cells favored a pro-inflammatory role by induction of various inflammatory cytokines [82]. Further studies failed to provide a clear picture for this adipokine in NAFLD. Surprisingly, in our study resistin serum levels increased 6 months after massive weight loss achieved by bariatric surgery but again fell below baseline values after 12 months [80]. Interestingly, weight loss in this patient cohort of severely obese subjects resulted in a significant decrease of hepatic mRNA expression of resistin. Several studies in the last years revealed divergent results with normal to elevated serum levels in NAFLD [83,84,85]. A single small study even demonstrated decreased resistin levels in the presence of NASH [86]. These results are challenged by another report investigating biomarkers of human NASH [87]. In this study, the authors studied biomarkers in 648 histologically evaluated NAFLD patients demonstrating that fibrosis was associated with increased serum levels of resistin, IL-8, monocyte chemoattractant protein 1, soluble IL-1 receptor type and TNFα. Similar increased levels and an association with fibrosis were observed in another recent study suggesting that resistin may probably reflect a pro-inflammatory profibrogenic adipokine in NAFLD [88]. In contrast to mice, monocytes/macrophages are considered as the major source for resistin which may point towards a different biology in rodents and humans. A more detrimental role for resistin in humans is suggested by other recent studies in type 2 diabetes patients where resistin levels correlated with overall mortality [89]. Despite these inconsistencies, one may conclude that increased resistin levels are associated with human pathologies rather conferring detrimental effect(s), especially in the case of advanced NASH [90,91].

### 4.3. Chemerin

Chemerin is a protein identified as the natural ligand of ChemR23 (chemerinR), an orphan G protein-coupled receptor expressed in immature dendritic cells and macrophages [92]. Both, adipose tissue and liver have been identified as a source of this adipokine [93]. We have observed that systemic chemerin levels decrease after successful bariatric surgery and this was paralleled by significant reduction of hsCRP levels [94]. This fits with several studies showing that NAFLD and disease severity correlated with increased chemerin levels [95,96]. Whereas some evidence exists that liver chemerin expression correlated with NAFLD features [96], others failed to show such an association [50,97]. In this study, hepatic chemerin expression did not relate to the degree of fibrosis and expression was even reduced in case of NASH. A lower visceral adipose tissue expression of chemerin was also observed in another NAFLD cohort [98]. Therefore, it currently remains unclear which role this adipokine plays in human NAFLD.

## 5. Conclusions

**Figure 1:**
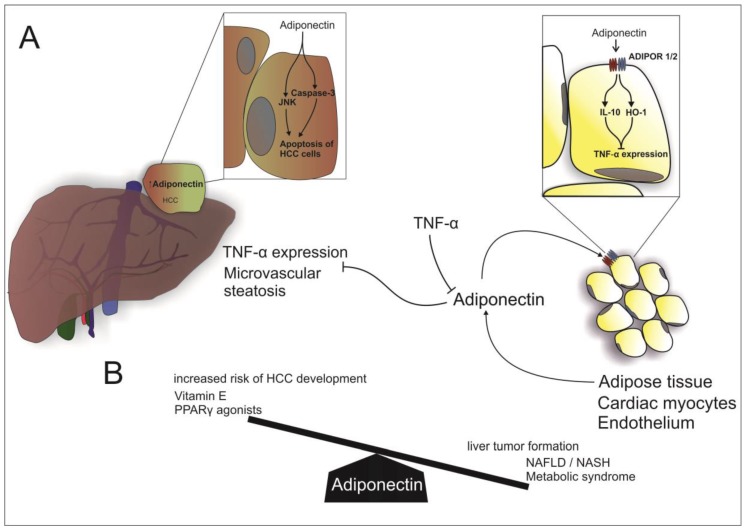
Adiponectin in NAFLD: (**A**) Beside cardiac myocytes and endothelial cells, adiponectin is mostly secreted by adipose tissue. Adiponectin acts in a paracrine manner via binding to ADIPOR 1/2 (Adiponectin receptor 1 and 2) and thereby inducing IL-10 (Interleukin 10) and HO-1 (Heme oxigenase-1), resulting in an inhibition of TNFα (tumor necrosis factor α) expression. Vice versa, TNFα dampens adiponectin transcription. Furthermore, adiponectin inhibits hepatic TNFα expression and microvascular steatosis. In HCC, adiponectin stimulates apoptosis of cancer cells by activation of Caspase 3 and JAK (Jun *N*-terminal kinase); (**B**) High levels of adiponectin are associated with an increased risk of HCC and the use of Vitamin E and Peroxisome proliferator-activated receptors γ agonists. On the other hand, low levels of adiponectin are associated with hepatic tumor formation, NAFLD, NASH and the metabolic syndrome.

**Figure 2:**
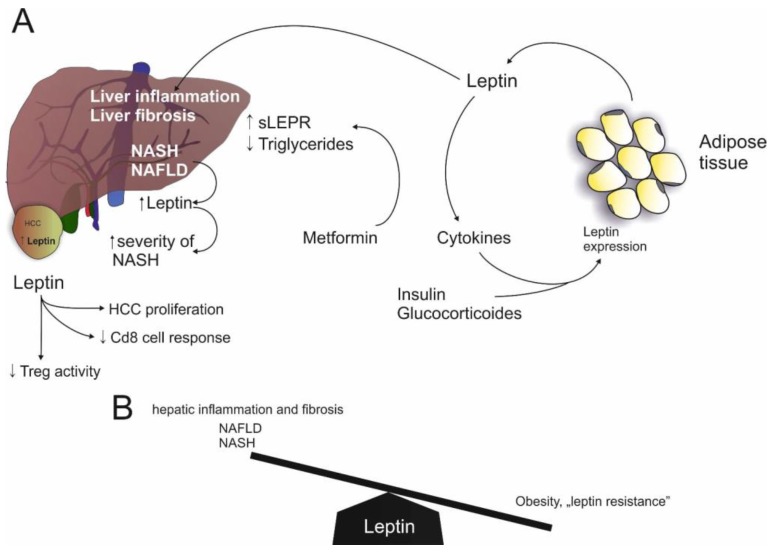
Leptin in NAFLD: (**A**) Leptin expression is induced by insulin, glucocorticoids and cytokines and leptin stimulates cytokine expression. Furthermore, leptin induces hepatic inflammation and fibrosis. Likewise, NAFLD and NASH are associated with increased leptin levels. On the other hand, Metformin induces sLEPR (soluble leptin receptor). In HCC, leptin induces proliferation but reduces CD8 response and Treg (regulatory T cell) activity; (**B**) Increased levels of leptin are associated with hepatic inflammation and fibrosis, as seen in NAFLD and NASH. On the other hand, low leptin levels are associated with leptin resistance in obesity.

In the last 15 years, researchers have gained substantial insights in the role of adipose-tissue derived mediators in health, obesity and obesity-related disorders. Whereas the role of prototypic adipokines such as adiponectin or leptin in NAFLD is rather established (Figure 1 and Figure 2, and Table 1), the impact of many other adipokines is still unclear and paralleled by substantial conflicting findings, especially with respect to resistin and NAMPT. Despite this fact, it appears established that adipose tissue (both subcutaneous and visceral) plays a fundamental role in systemic inflammatory processes associated with obesity. Evidence for a dominant role of adipose tissue in NAFLD is accumulating [5,99]. Recent studies have convincingly shown that adipose tissue with its enormous content of mediators communicates with metabolically active organs beyond the liver [100,101]. As such, visceral adipose tissue, a cytokine sink, contains an enormous amount of systemically active mediators such as adipokines and cytokines. These mediators contribute to low-grade inflammation observed in severe obesity and associated disorders such as NAFLD and might even play a role in the most extreme complication of this disorder, i.e., cancer development. We have to acknowledge that human studies investigating either serum/plasma levels or tissue expression of a certain adipokines remain simply descriptive with limited insights. Only careful preclinical and clinical studies will allow additional insights and, hopefully, establish the basis for future therapeutic interventions.

## Figures and Tables

**Table 1:** Adiponectin and Leptin in NAFLD and HCC.

	NAFLD	HCC
Adiponectin	Decreased plasma levels in obese subjects and patients with insulin resistance Decrease in circulating adiponectin in lean NAFLD patients Decreased levels in blood and liver tissue in NASH Increase of hepatic expression after weight loss Protective metabolic player	Increased serum levels: Higher risk of HCC development Poor HCC survival Increased serum Adiponectin levels in HCC Hypoadiponectinemia promotes experimental liver tumor formation Inverse correlation of adiponectin expression in tissue with tumor size Non-HMW adiponectin an attractive biomarker for predicting later development of HCC
Leptin	Mediates adipose-brain communication Steady state: improvement of metabolic dysregulation Obesity: failing to correct hyperglycemia ➔ “leptin resistance” Increase associated with liver disease severity Stable expression in liver tissue after weight loss Decrease in serum leptin levels after bariatric surgery Direct correlation with NAFLD severity (NASH and simple steatosis)	Increased serum Leptin levels in HCC Increased leptin expression in HCC samples Association with HCC proliferation in human HCC samples Mutation in LEPR increase susceptibility to hepatocarcinogenesis

## Abbreviations

ADIPOR	Adiponectin Receptor
AFP	α-fetoprotein
AMP	Adenosine monophosphate
AMPK	5′ AMP-activated protein kinase
BCLC	Barcelona clinic liver cancer
CD	Cluster of differentiation
chemerinR	chemerin Receptor
CHIP	*C*-terminus of Hsc70-interacting protein
CXCL	Chemokine (C–X–C motif) ligand
FGF	Fibroblast growth factor
FOXO	Forkhead box O
HCC	Hepatocellular carcinoma
HFD	High-fat diet
HMW	High molecular weight
HO-1	Heme oxygenase 1
hsCRP	High-sensitivity C-reactive protein
IL	Interleukin
JAK	Janus kinase
JNK	c-Jun *N*-terminal kinase
KO	knockout
LEPRb	Leptin receptor type B
MMP	Matrix metalloproteinase
NAD	Nicotinamide adenine dinucleotide
NAFLD	Non-alcoholic fatty liver disease
NAMPT	Nicotinamide phosphoribosyltransferase
NASH	Non-alcoholic steatohepatitis
*ob*/*ob*	Leptin-deficient
PBEF	pre-B-cell colony-enhancing factor
STAT	Signal transducer and activator of transcription
SIRT	Sirtuin
TNFα	Tumor necrosis factor α
PPARγ	Peroxisome proliferator-activated receptor γ
